# Episodic memory and semantic knowledge interact to guide eye movements during visual search in scenes: Distinct effects of conscious and unconscious memory

**DOI:** 10.3758/s13423-025-02686-6

**Published:** 2025-05-21

**Authors:** Michelle M. Ramey, John M. Henderson, Andrew P. Yonelinas

**Affiliations:** 1 Department of Psychological Science, University of Arkansas, Fayetteville, AR, USA; 2 Department of Psychology, University of California, Davis, CA, USA; 3 Center for Mind and Brain, University of California, Davis, CA, USA; 4 Center for Neuroscience, University of California, Davis, CA, USA

**Keywords:** Memory, Semantic knowledge, Visual attention, Visual search, Eye tracking

## Abstract

Episodic memory and semantic knowledge can each exert strong influences on visual attention when we search through real-world scenes. However, there is debate surrounding how they interact when both are present; specifically, results conflict as to whether memory consistently improves visual search when semantic knowledge is available to guide search. These conflicting results could be driven by distinct effects of different types of episodic memory, but this possibility has not been examined. To test this, we tracked participants’ eyes while they searched for objects in semantically congruent and incongruent locations within scenes during a study and test phase. In the test phase containing studied and new scenes, participants gave confidence-based recognition memory judgments that indexed different types of episodic memory (i.e., recollection, familiarity, unconscious memory) for the background scenes, then they searched for the target. We found that semantic knowledge consistently influenced both early and late eye movements, but the influence of memory depended on the type of memory involved. Recollection improved first saccade accuracy in terms of heading towards the target in both congruent and incongruent scenes. In contrast, unconscious memory gradually improved scanpath efficiency over the course of search, but only when semantic knowledge was relatively ineffective (i.e., incongruent scenes). Together, these findings indicate that episodic memory and semantic knowledge are rationally integrated to optimize attentional guidance, such that the most precise or effective forms of information available – which depends on the type of episodic memory available – are prioritized.

## Introduction

If we typically keep our coffee mug on our desk, we can rely on semantic knowledge for its usual location to find it later. Given this, do we still leverage episodic memory for the specific location in which we last placed our mug? In the present study, we examined the influence of episodic memory on visual search when guidance by semantic knowledge is also available – and how these two sources of attentional guidance interact.

Semantic knowledge for spatial statistical regularities in the world, such as how objects tend to co-occur, is highly effective at guiding attention during visual search through scenes (Neider & Zelinsky, 2006; Peacock et al., 2021; Pereira & Castelhano, 2019; Sefranek et al., 2024; Torralba et al., 2006; Wu, Wang, et al., 2014a, Wu, Wick, et al., 2014b). In addition, newly learned information in the form of episodic memory is also effective at guiding search (Becker & Rasmussen, 2008; Brockmole & Henderson, 2006; Chun, 2000; Chun & Jiang, 1998; Goujon et al., 2012; Kit et al., 2014; Li et al., 2016, 2018; Summerfield et al., 2006). It is not clear, however, how these two forms of attentional guidance influence search when both are present.

Studies examining this issue typically ask participants to search for objects in scenes. Some find that memory has very little influence on search performance when semantic knowledge is available to guide search – that is, in standard real-world scene contexts where objects follow expected semantic relationships (Oliva et al., 2004; Võ & Wolfe, 2012; Wolfe et al., 2011). This effect is typically interpreted as indicating that memory is slower to access or deploy than immediately available scene information such as the scene’s semantic properties; thus, semantic knowledge is prioritized above memory to guide attention more efficiently (O’Regan, 1992; Võ & Wolfe, 2013). Others using similar tasks, however, find that memory robustly improves search even when semantic knowledge is effective – leading to the conclusion that memory consistently guides search even when semantic knowledge is available (Goujon et al., 2012; Hollingworth, 2009, 2012; Howard et al., 2011; Li et al., 2018; Summerfield et al., 2006). The reasons for these discrepancies are not yet clear.

Despite the points of contention, there is one finding that is agreed upon: Even when semantic knowledge is available, memory that is formed by searching for a target substantially improves later search performance for that target. The point of contention is whether memory for objects that were *not* previously searched for (e.g., distractor objects) can substantially improve later search performance for those objects – even though it is generally agreed that the objects are indeed encoded (Castelhano & Henderson, 2005). What differs between a previously searched-for object and an incidentally viewed object? We propose that a critical difference is the type or strength of memory that was formed. In fact, it is known that searching for objects produces stronger memories for those objects than do other types of encoding (Draschkow et al., 2014; Helbing et al., 2020). Despite this, the role of memory strength or type during semantically relevant search has not been examined.

Recent research indicates that different types of memory exert distinct effects on semantically irrelevant search. Specifically, episodic memory can be supported by vivid, detailed *recollection*; gist-like *familiarity*; or *unconscious memory* outside of awareness^1^ (Yonelinas et al., 2022; Zimmer & Ecker, 2010). During search for letters arbitrarily embedded in scenes, unconscious memory was found to gradually improve the eyes’ scanpath efficiency (Ramey et al., 2019), in line with proposals that memory is slow to guide search. In contrast, recollection was found to guide the first eye movement (i.e., first saccade) more directly towards the target. Critically, this indicates that memory can be slow or rapid in guiding search, depending on the type(s) of memory available.

The fact that different types of memory contribute to search via distinct attentional patterns has important implications for how semantic knowledge and memory may influence search when both are present. First, this suggests that conflicting findings on the extent to which memory influences semantically relevant search could be driven by task-related differences in the type(s) of memory available. A second, broader implication beyond extant debates is that different types of memory could differentially interact with semantic knowledge. These possibilities have yet to be examined.

### Current research

To address these questions, we tracked participants’ eye movements while they searched for objects in scenes during a study and test phase. We manipulated the effectiveness of semantic guidance via schema congruency: Target objects were located in schema-congruent locations (e.g., toothbrush by the sink) or schema-incongruent locations (e.g., toothbrush on the toilet). During the test phase, participants made recognition responses to the background scenes (without the target) before searching for the target. We examined how congruency interacted with different memory processes to influence search-related eye movements.

We expected to conceptually replicate Võ and Wolfe’s (2013) findings in which memory-driven search improvements were larger when semantic knowledge was less effective (i.e., scrambled object arrays). Specifically, we expected to find larger memory-driven search improvements for incongruent scenes (i.e., scenes in which semantic knowledge is less effective) than congruent scenes. As for memory processes, given that there is no prior work to our knowledge examining how different memory processes interact with semantic knowledge during search, we partially based our predictions on work examining interactions between memory and semantic knowledge in spatial recall of target object locations. Specifically, the typically robust influence of semantic knowledge on spatial recall is greatly reduced when recollection is available (Ramey et al., 2022; Ramey & Zabelina, 2024). Based on this, along with findings that recollection influences early eye movements (Ramey et al., 2019), we expected that recollection would compete with semantic knowledge to guide early eye movements such that recollection should reduce the effects of schema congruency. As for familiarity, we did not expect it to influence search given prior findings that familiarity did not guide search (Ramey et al., 2019).^2^ Based on Võ and Wolfe’s (2013) proposal that the visual stimulus (including its semantic properties) is prioritized in guiding search due to memory being relatively slower to access, we predicted that unconscious memory – previously shown to be relatively slow to guide search (Ramey et al., 2019) – would primarily influence performance after semantic knowledge has failed. That is, we predicted that unconscious memory would “kick in” after initial semantically guided eye movements. Because these early semantically guided eye movements should lead to successful search completion in congruent scenes – therefore ending search before unconscious memory processes could take effect – we expected that unconscious memory would primarily influence performance in incongruent scenes.

## Method

### Participants

Forty undergraduate participants completed the experiment for course credit. A sample size of 35 participants was needed to provide 99% power to detect the smallest analogous effect in a prior eye-tracking study (i.e., the effect of recollection on first saccade accuracy; Ramey et al., 2019), using *d*_z_=.75 in a two-tailed paired-samples t-test. The quality of each participant’s eye-tracking data was assessed by computing the mean percent signal across all trials to determine whether there was excessive track loss due to blinks or calibration loss, with a preselected criterion of 75% signal (Henderson & Hayes, 2017). All but one participant had greater than the signal criterion (for the remaining participants *M* = 97.7%; range = 87.7–99.9%), so data from the remaining 39 participants were retained for analysis.

### Apparatus

Participants’ eye movements were recorded using an SR Research EyeLink 1000+ tower mount eyetracker, sampling at 1,000 Hz. Stimuli were displayed on a 21-in. monitor 85 cm from participants’ eyes at a resolution of 1,024 × 768 pixels.

### Materials

Stimuli were 130 photographs of real-world scenes presented in color. Five scene categories were used, and a single type of target object was used for each category. The categories and targets consisted of kitchens (target: frying pan), dining rooms (target: wine glass), bedrooms (target: alarm clock), living rooms (target: coffee mug), and bathrooms (target: toothbrush cup). Eight different object exemplars were used per category, such that the visual features of the target object varied across different scenes within a category. In each scene, only one exemplar of the target object was present, and this was kept consistent across presentations. For example, in each living room scene, there was only one coffee mug present. Importantly, for a given scene viewed by a given participant, the target was always visually identical and in the same location across repeated viewings.

**Fig. 1:**
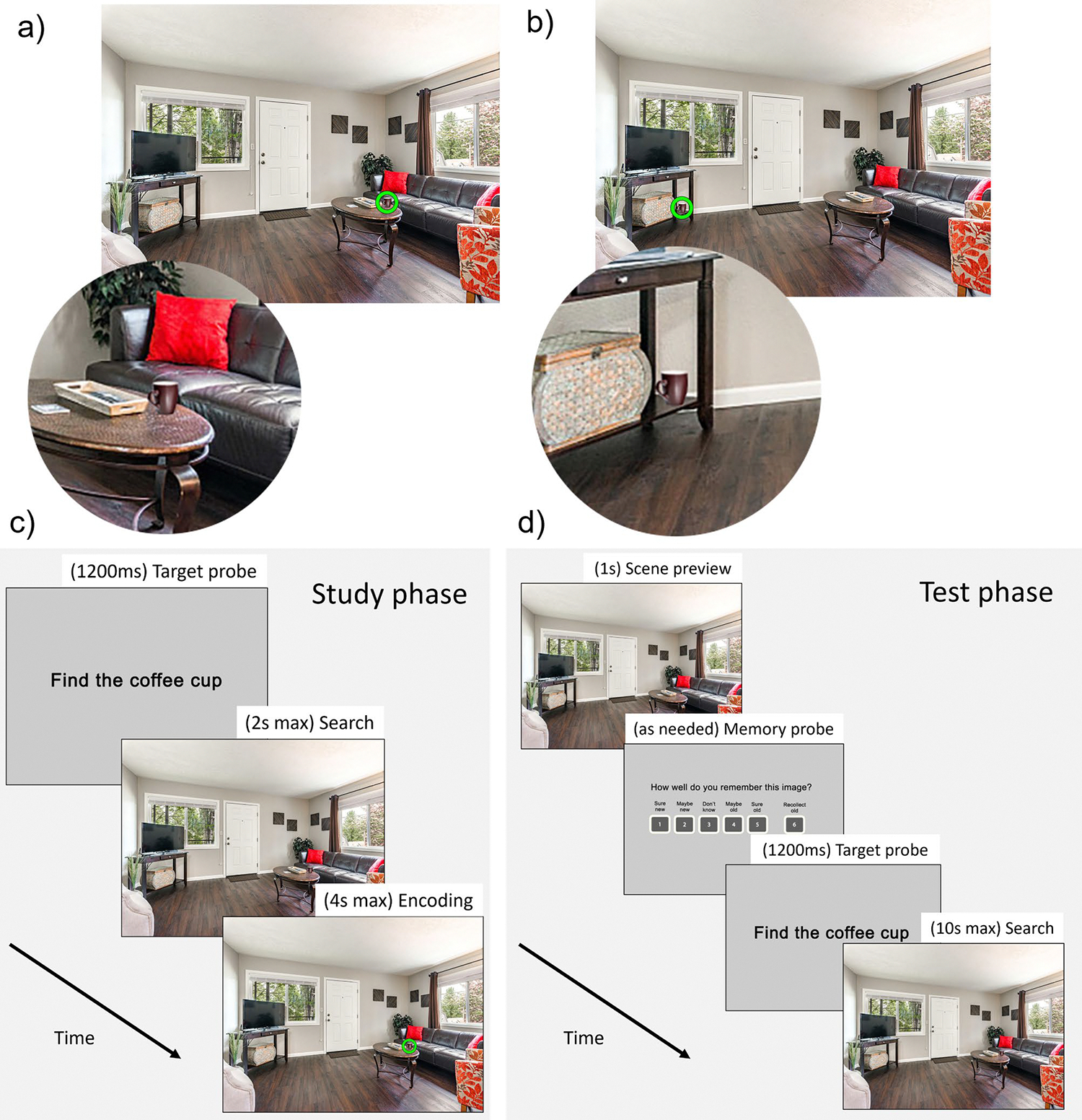
Stimuli and procedure. (**A**) Congruent scene, with closeup of target for illustration. (**B**) Incongruent scene, with closeup of target for illustration. (**C**) The trial sequence in the study phase. In each trial, a target probe appeared (e.g., “Find the coffee cup”), followed by a fixation cross (not shown), followed by the scene with target object. Participants were required to search for and fixate the target object within 2 s. After fixating the target or after 2 s, whichever occurred first, a ring appeared around the target to afford participants additional time to encode the scene and target location. The duration of this post-search encoding phase was dynamically determined such that all scenes were displayed for a total of 4 s per trial (distributed across both search and encoding). (**D**) The trial sequence in the test phase. In each trial, participants first saw a fixation cross, then a preview of the scene without the target object. A memory probe then appeared, and participants gave a confidence-based recognition memory response for the scene. After responding, a fixation cross was shown, followed by the same scene but with the target now present. Participants were given 10 s to search for the target and press the spacebar when they located it

Two versions of each scene were created using Adobe Photoshop (Figs. 1a, b): one with the target object in an expected location based on scene semantics (i.e., schema-congruent scene), and one with the target in an unexpected location (i.e., schema-incongruent scene). Importantly, photoshop was used to place the object in every scene, so any visual saliency effects introduced by photoshop did not differ systematically between congruent and incongruent scenes. The congruent location was consistent across all scenes in a category, such that targets were placed relative to larger objects (i.e., anchor objects) with which the target objects co-occur with high probability in daily life (Boettcher et al., 2018). It is well established that semantic knowledge guides eye movements to schema-congruent locations during search (Boettcher et al., 2018; Torralba et al., 2006; Wynn et al., 2020); thus, the congruency conditions manipulated the extent to which semantic knowledge would improve or interfere with search. For the congruent condition, in bathroom scenes, the toothbrush cups were located on counters near sinks; in dining room scenes, the wine glasses were on tables; in kitchen scenes, the pans were on stove burners; in bedroom scenes, the alarm clocks were on nightstands within arm’s reach of the bed; and in living room scenes, the coffee mugs were on coffee tables. In incongruent scenes, on the other hand, the objects were arbitrarily placed in unexpected but physically plausible locations (i.e., on floors, shelves, chairs, etc. rather than floating).

Scene congruency was manipulated within-subjects such that each participant was presented with half incongruent scenes and half congruent scenes. The congruent and incongruent versions of the scenes were counterbalanced such that half of the participants saw the congruent version of a given scene, whereas the other half saw the incongruent version of that same scene. Importantly, a given scene was always congruent or incongruent within a given counterbalance (given that the target was always in the same place in a scene viewed across multiple repetitions by a given participant).

To validate the congruency manipulation, we also had a separate group of participants (*n* = 25) rate each scene for the congruency of its target object. Specifically, participants were asked to rate how normal or unusual a target’s location was on scale of 1–6. These scores were averaged together to produce a congruency score for each scene. As expected, congruent scenes were rated as significantly more congruent than were incongruent scenes, *p* < .001.

### Procedure

The experiment lasted approximately 70 min and consisted of a study phase followed by a test phase (Figs. 1c, d). There was a 5-min break between the study and test phases. Participants were also given breaks every 50 trials. The eye tracker was recalibrated after each break. Before each phase, participants were given instructions as well as practice trials to familiarize them with the procedure. All procedures were approved by the University Institutional Review Board.

### Study phase

Participants were told that they would be searching for target objects and were asked to try to remember the scenes for a later memory test. During the study phase, participants were presented with 100 unique scenes that were each presented twice, for a total of 200 trials. The repetitions were randomly intermixed throughout the study phase, with the requirement that the same scene did not appear twice in a row. In each trial, participants were first given a 1,200-ms probe alerting them to the target object they would need to search for (Fig. 1c). Then, a fixation cross appeared that lasted a minimum of 600 ms, and the trial proceeded once they fixated the fixation cross. The scene then appeared, and participants had 2 s to search for and fixate the target object in the scene. This duration was determined based on initial piloting; in the present data, participants successfully fixated within 50 px of the target on 86% of study phase trials. After fixating the object, or after 2 s elapsed, a ring appeared around the target object to allow participants additional time to encode the scene and target location. If they had found the target object within 2 s, the ring was green; if they had not found the target object within 2 s, the ring was red. The duration for which the ring and scene remained onscreen after search was dynamically determined such that all scenes were displayed for a total of 4 s per trial including both the search time and the post-search encoding time. This procedure ensured that search difficulty could not influence the total time that the scene and target object were displayed during the study phase.

### Test phase

In the test phase, participants were asked to provide a confidence-based recognition memory judgment for each scene, and to search for the target object in each scene. As in prior work (Ramey et al., 2019), at the beginning of each trial before search commenced, participants were presented with a 1s preview of the scene without the target in it. Participants were asked to make a recognition judgment based on the preview. Specifically, memory strength was measured by asking participants to rate memory confidence for each scene on a 6-point scale during the recognition judgment (Yonelinas, 2002). Note that participants’ memory confidence for the background scene was probed, not their confidence in their memory for the target location. We probed memory for the background scene to provide an index of underlying memory for the episode that was free from contamination by influences of schema congruency available at retrieval (Ramey et al., 2024). Participants were told that if they could consciously recollect some qualitative aspect of the initial learning event, such as what they thought about when the scene was encountered earlier, they should respond “Recollect old (6);” otherwise, they rated their memory confidence by responding “I’m sure it’s old (5),” “Maybe it’s old (4),” “I don’t know (3),” “Maybe it’s new (2),” or “I’m sure it’s new (1).” Importantly, participants were instructed that a “sure old” response was equal in confidence to a “recollect old” response, such that the only difference between them was that at least one specific detail of the learning event was remembered in recollected scenes. Participants were instructed and tested on how to use this scale, with particular focus on ensuring they understood the difference between “sure old” and “recollect old,” prior to beginning the test phase.

After the recognition judgment, the trial proceeded much like the study phase search trials. Participants were given a 1,200-ms probe alerting them to the target object they would need to search for, then a fixation-contingent fixation cross appeared, then the scene was presented again, this time with the target present. Participants were given 10 s to search for the target, which was found in 99.8% of trials. They were asked to press the spacebar when they found the target, which terminated the trial. The test phase included 130 scenes, 100 of which were presented in the study phase and 30 of which were new lures. Each scene was presented once, for a total of 130 test trials. We included more old scenes than new scenes to ensure that an adequate number of old scenes was recognized at each level of confidence for analysis. In particular, we needed an adequate number of high-confidence missed old scenes (“sure new”) in order to assess unconscious memory effects. (Note that our analytic method does not require equal trial numbers across old and new scenes, nor equal trial numbers in different response bins.) Stimulus presentation was counterbalanced, such that the scenes appeared in different conditions (i.e., presented at both study and test, or used as a new lure during test) for different participants to mitigate stimulus effects.

### Data reduction and analysis

**Fig. 2:**
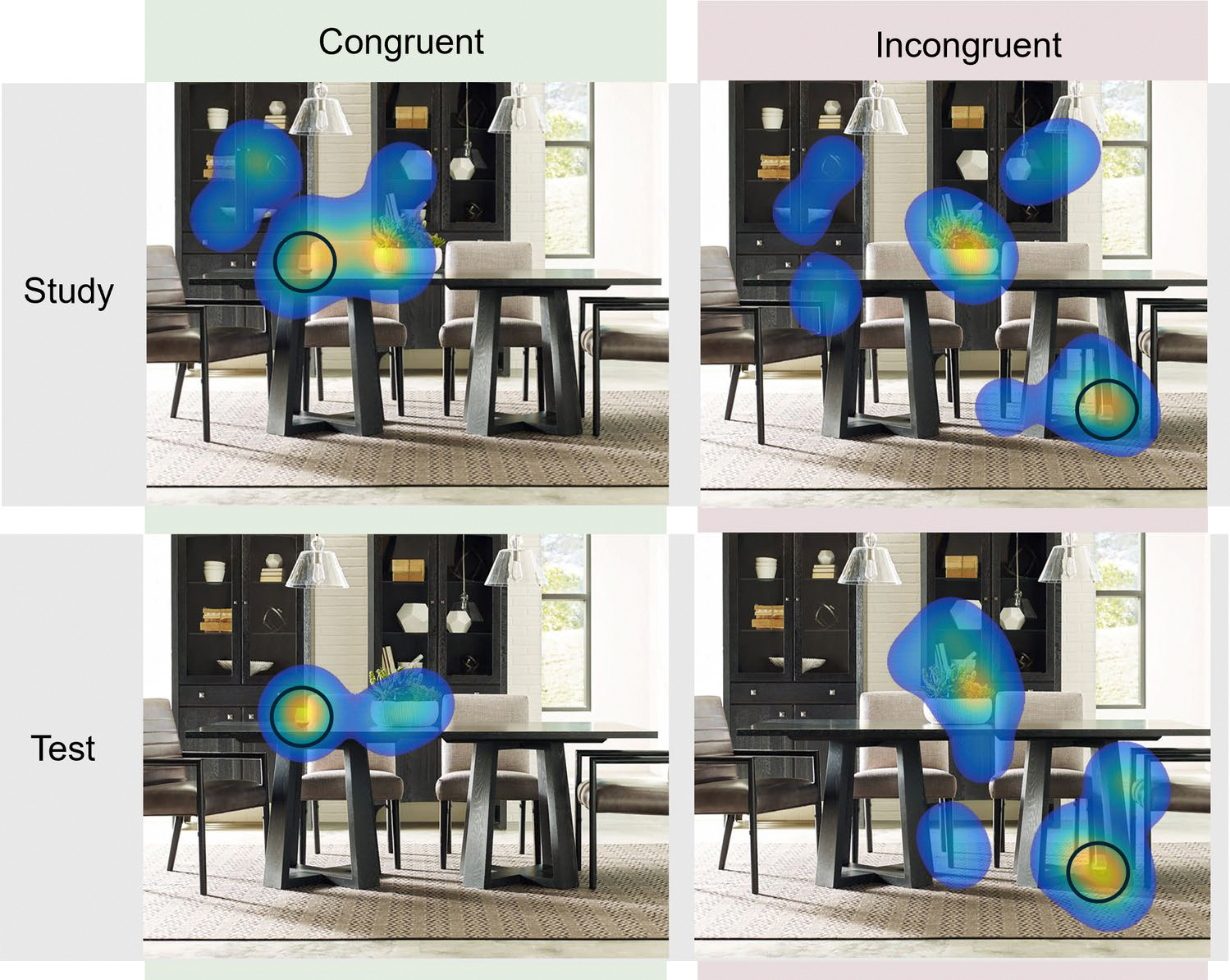
Heatmaps of participants’ fixations on a sample scene for each condition. To illustrate effects of memory, the test scenes only include fixations from when the scenes were old. Brighter regions indicate a higher density of fixations. The target object (wine glass) is circled for illustration. To aid in visualizing the general trends across participants, isolated fixations (i.e., individual fixations that were not grouped with any other fixations) were removed

The fixation heatmaps for a sample scene, separated by congruency and study versus test, are presented in Fig. 2. Analyses were conducted on test-phase data only, in which search was preceded by recognition judgments. Trials were excluded from analysis if participants did not respond (using the spacebar) that they had found the target, if participants responded but were not recorded as fixating within 3 degrees of visual angle (125 px) of the target, or if participants only made one fixation in a trial. (However, note that the results are unchanged when all trials, regardless of response or fixation status, are included.)

We examined eye-movement metrics of search performance based on prior memory-guided search findings (i.e., dissociable memory effects on early eye movements versus efficiency over the course of a trial; Ramey et al., 2019), as well as eye-movement metrics aimed at directly examining guidance by semantic knowledge (i.e., viewing of congruent regions; Wynn et al., 2020).

For eye-movement metrics indexing search performance (i.e., first saccade accuracy and scanpath efficiency, described below), we analyzed both the raw measures as well as a corrected version of the measures to control for potential effects of scaling. Specifically, an inherent consequence of congruent scenes is that search performance is typically closer to ceiling than incongruent scenes, leading to scaling differences between congruent and incongruent scenes resulting from differences in baseline performance. For example, if memory improved performance in all scenes by 20%, this would produce a much larger improvement to incongruent than congruent scenes in terms of raw performance metrics. Note, however, that performance in the present study was not at ceiling: The median number of fixations occurring prior to initial target fixation was four for congruent scenes. Nevertheless, to examine potential effects of scaling, we also report analyses using a rescaled version of our measures that represents the proportion improvement from baseline performance (i.e., performance in new scenes) for old scenes in that congruency condition. That is, the score (e.g., first saccade accuracy) for each old scene was divided by the average score in new scenes for its respective congruency condition.

### First saccade accuracy

**Fig. 3:**
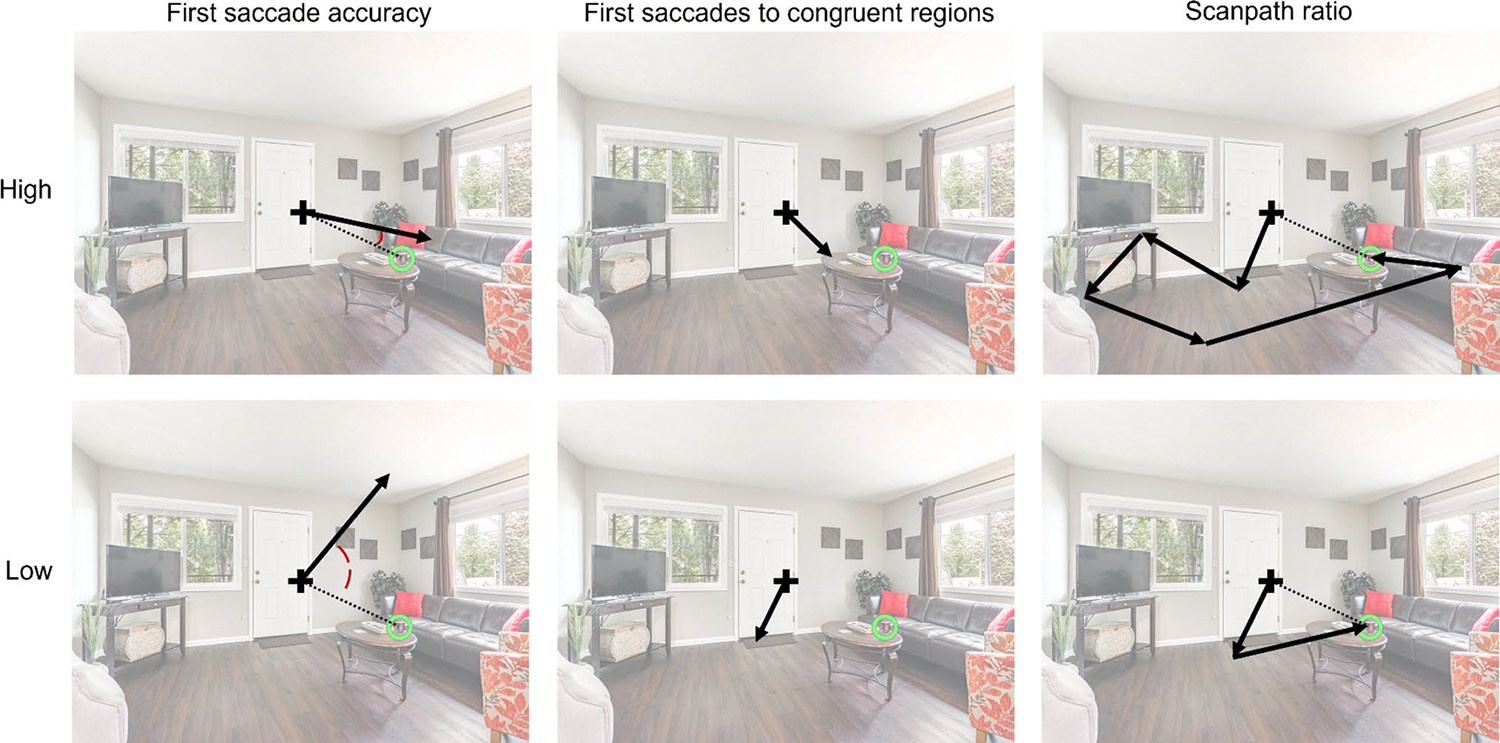
Examples of high and low values of each eye-movement measure on a sample scene. The arrows represent saccades; for the measures of first saccade, the arrows represent the first saccade. The dashed line represents the direct (i.e., ideal) path to the target, which served as the comparison for computing first saccade accuracy and scanpath ratio. For first saccade accuracy, the only relevant aspect of the saccade was the direction in which it was pointed. For first saccades to congruent regions, the only relevant aspect of the saccade was whether it landed on a congruent region. For scanpath ratio, the only relevant aspect of the saccades was their total distance. Note that the fixation cross, and ring highlighting the target object, are shown only for illustrative purposes. There was no fixation cross or ring present while participants were searching; the fixation cross was only present prior to search scene onset. The opacity of the scene was lowered for ease of visualizing the measures

We assessed the extent to which the first saccade in a trial (starting from the center fixation cross) was aimed towards the target after search scene onset (Fig. 3). To do this, we calculated the angular distance, in terms of degree error, between the saccade and the most direct path to the target. The direct path to the target was defined as the path from the starting point of the saccade (i.e., a participant’s actual fixation location on the center cross upon search scene onset) to the target. Degree error ranged from 0 to 180 degrees, with higher values indicating lower accuracy. Note that the saccade length was not relevant to this measure – only the direction of the saccade. (Scanpath ratio below accounts for distance of saccades.) Degree error was skewed and it was therefore log-transformed for statistical analyses; for the sake of interpretability, untransformed data are presented for plots as well as for the reported values of percentage changes when correcting for scaling.

### First saccades to congruent regions

We also assessed whether the first saccade upon search scene onset landed on a congruent region (Fig. 3). Congruent regions were defined as the surfaces on which a given target object is expected to be located based on semantic knowledge and surface information (Boettcher et al., 2018; Draschkow & Võ, 2017; Peacock et al., 2021; Võ & Henderson, 2009). Specifically, congruent regions encompassed the entire surface of the object(s) on which the target objects are located with high probability in daily life (see Materials section for specific pairings of target and anchor objects). When fixations were located in these regions, they were classified as congruent fixations.

### Scanpath ratio

We assessed the efficiency of attentional guidance over the course of each trial using scanpath ratio (Fig. 3), which compared the total distance travelled by the eyes to the shortest possible path (Castelhano & Henderson, 2007). Specifically, the total scanpath length in each trial was divided by the distance between the starting fixation and the center of the target, with lower values indicating a more efficient search. Scanpath ratio was skewed and it was therefore log-transformed for analysis; for the sake of interpretability, untransformed data are presented for plots as well as for the reported values of percentage changes when correcting for scaling.

### Statistical models

All analyses used data from the test phase. Statistical analyses were conducted using multilevel modeling, which allowed us to harness trial-by-trial (i.e., within-subjects) data while controlling for individual differences and stimulus-specific effects. To do this, we used crossed random intercepts of subject and image for all analyses, which also accounted for the dependencies between trials. We also tested random slopes for each analysis – allowing the predictor(s) of interest to vary by subject and image – but found that they produced singular fits in almost all cases, indicating that the models were overparameterized with slopes included. However, the results were unchanged when random slopes were included: Null results remained null, and significant effects remained significant. The dependent variable in each model was the eye movement measure of interest. Depending on the specific analysis, as specified in the Results section, the fixed variables of interest could be number of scene presentations (i.e., old vs. new scenes), memory response, and/or congruency condition. Continuous predictors were scaled. When the outcome of analysis was continuous, linear mixed effects models were used (Kuznetsova et al., 2017); effect sizes are reported as β for continuous fixed effects and standard mean difference (*SMD*) for categorical fixed effects. When the outcome of analysis was binary, logistic mixed effects models were used, and effect sizes are reported as *B*. (Note that all reported effect sizes simply reflect the model estimate.) The model equations are provided in the Online Supplementary Materials (OSM) and referenced throughout the results (Eqs. S1–S5).

To quantify the evidence in favor of the null for key non-significant results (with continuous outcomes) that were obtained using frequentist statistics, we computed Bayes factors (using the *BayesFactor* package in R with 5000 iterations) and assessed B F_10_ – that is, the ratio of the Bayes factor in favor of the alternative hypothesis to the Bayes factor in favor of the null hypothesis. By convention, a B F_10_ < 0.33 indicates moderate evidence for the null hypothesis, and a BF_10_ < 0.10 indicates strong evidence for the null (Lee & Wagenmakers, 2013).

We separately examined recollection, familiarity strength, and unconscious memory in different models. We assessed effects of recollection-based memory by comparing performance between recollected scenes (i.e., scenes that participants endorsed as “I recollect it’s old”) and familiar scenes that were matched in confidence (i.e., scenes that participants endorsed as “I’m sure it’s old”).

We examined effects of familiarity strength by comparing performance across the continuum of familiarity responses, including all response bins ranging from no familiarity (“I’m sure it’s new”) to highest strength familiarity (“I’m sure it’s old”).

Lastly, we examined effects of unconscious memory by comparing performance between new scenes (containing no memory of any kind) and old scenes that participants endorsed as “I’m sure it’s new,” such that they had no conscious memory awareness for the scenes despite having seen them previously. (Note that all results below were equivalent regardless of whether the new scenes in this unconscious comparison included all new scenes, or only “I’m sure it’s new” new scenes.) Any performance differences between new scenes and “I’m sure it’s new” old scenes indicate unconscious memory effects – that is, experience-driven behavioral changes that occur despite a confident lack of awareness for that experience. (Note that this behavioral analysis speaks to effects of awareness of memory, but cannot speak to differences in underlying memory representations.) Importantly, the presence of an unconscious memory effect does not necessitate that performance varies across memory responses. In fact, a selective unconscious memory effect (i.e., an unconscious memory effect without additional effects of familiarity or recollection) on an eye-movement metric would produce results in which the eye-movement metric does not vary across memory response at all; for a selective unconscious memory effect, differences in the eye-movement metric would only arise when comparing old scenes with new scenes. That is, for an eye-movement measure that is only influenced by unconscious memory, participants’ memory response becomes irrelevant. However, it is also possible that performance can be influenced by multiple types of memory (as in Ramey et al., 2020, 2022), in which case eye-movement metrics would vary with response, in addition to an unconscious effect being observable in high-confidence misses.

## Results

Recognition performance, in terms of AUC, was well above chance, *t*(38) = 19.9, *p* < .001 (AUC = .75); memory response counts are presented in Table 1. As expected, both semantic knowledge and episodic memory contributed to search performance in terms of search speed, and we conceptually replicated the finding from Võ and Wolfe (2013) of more memory-driven improvements when semantic knowledge was less effective. Specifically, search speed in terms of time to fixate the target in the test phase was faster for congruent than incongruent scenes, *SMD* = −.34, *p* < .0001, and faster for old than new scenes, SMD = −.27, *p* < .0001 (Eq. S1a). There was an interaction such that the memory-driven improvements were larger in incongruent scenes, *SMD* = −.26, *p* < .0001 (Eq. S1b). Additional search speed analyses by memory type are presented in the Online Supplementary Materials.

### Effects on the first eye movement

#### First saccade accuracy

**Fig. 4:**
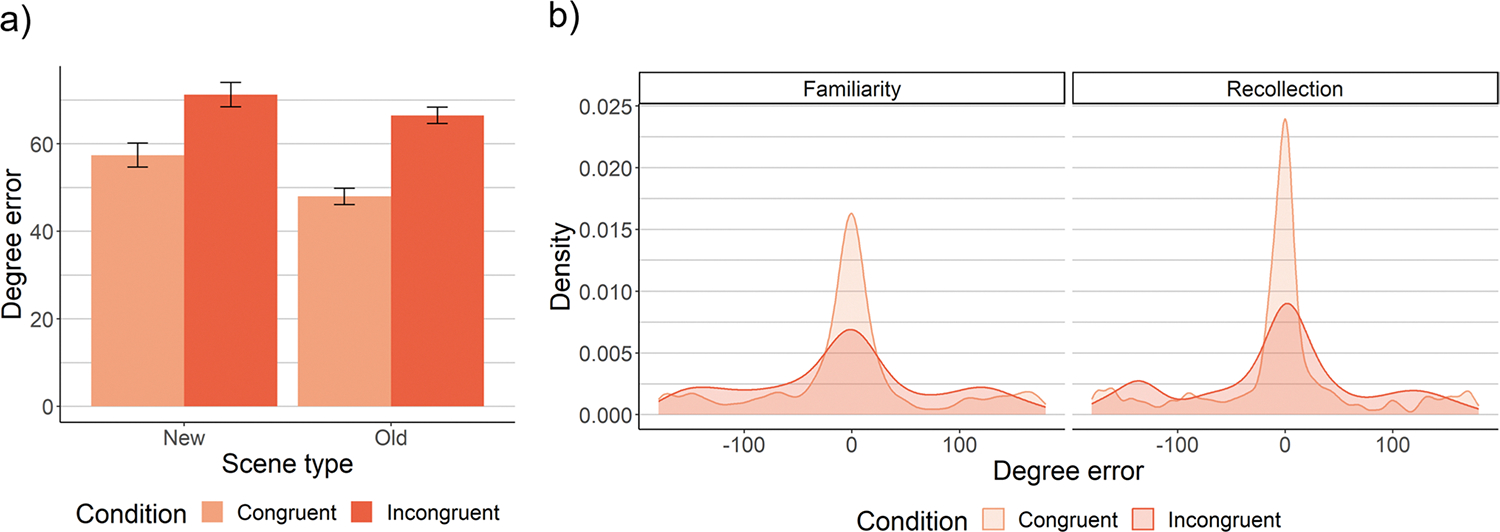
Effects of memory and semantic knowledge on first saccade accuracy. First saccade accuracy is the extent to which the first saccade off the central starting point was aimed towards the target; higher degree error values indicate lower accuracy. Overall memory improved first saccade accuracy for both congruent and incongruent scenes, and this effect was primarily driven by recollection. Specifically, recollection produced significantly better first saccade accuracy than high-confidence familiarity in both congruent and incongruent scenes. (**A**) First saccade accuracy for old versus new scenes by congruency condition. The estimated marginal means derived from a linear mixed effects model with random effects of scene and subject are plotted, and the error bars represent the standard error of these estimated means from the model. (**B**) Density plots of the distribution of first saccade accuracy. Familiarity includes responses of “sure old”, which are matched to recollection in terms of subjective strength. (Note that absolute error values from 0 to 180 were used for analysis)

We first examined the extent to which the first saccade was directed towards the target (Fig. 4a). Both memory and semantic knowledge improved first saccade accuracy, such that degree error was lower in old than new scenes, *SMD =* −.15, *p <* .0001 (Eq. S1a), and in congruent than incongruent scenes, *SMD =* −.39, *p <* .0001. Semantic knowledge and memory did not interact, *SMD =* .08, *p =* .20, BF_10_ = .11 (Eq. S1b), in contrast to theories that memory primarily influences search when semantic knowledge is ineffective.

As predicted, the effect of memory on first saccade accuracy was driven by recollection, *SMD =* −.12, *p =* .021 (Eq. S2a), rather than familiarity, *p*s > .42, BF_10_s < .07 (Eqs. S3), or unconscious memory, *p*s > .14, BF_10_s < .13 (Eqs. S4). That is, first saccade accuracy was higher for recollection than high-confidence familiarity (Fig. 4b), but there was Bayesian evidence that first saccade accuracy did not vary significantly across familiarity responses or between new scenes and forgotten old scenes. Recollection did not interact with congruency to influence first saccade accuracy, *SMD =* .001, *p =* .99, BF_10_ = .09 (Eq. S2b), such that recollection-driven improvements were similar for congruent and incongruent scenes. Interestingly, the fact that recollection improved first saccade accuracy, but did not interact with congruency to do so, suggests that recollection did not compete with semantic knowledge.

One potential reason for a lack of interaction between congruency and memory is that scaling differed between congruent and incongruent scenes – that is, baseline performance for congruent scenes was much better than incongruent scenes, *SMD =* −.39, *p <* .0001. Correcting for scaling, we found that memory improved first saccade accuracy by a higher proportion in congruent scenes, 17.4%, than incongruent scenes, 7.6%, *SMD =* −.11, *p =* .0007 (Eq. S5). Intriguingly, rather than competing, memory and semantic knowledge instead appeared to exhibit synergistic effects on the first eye movement: Memory-driven improvements were proportionally larger when semantic knowledge was also effective.

#### First saccades to congruent regions

**Fig. 5:**
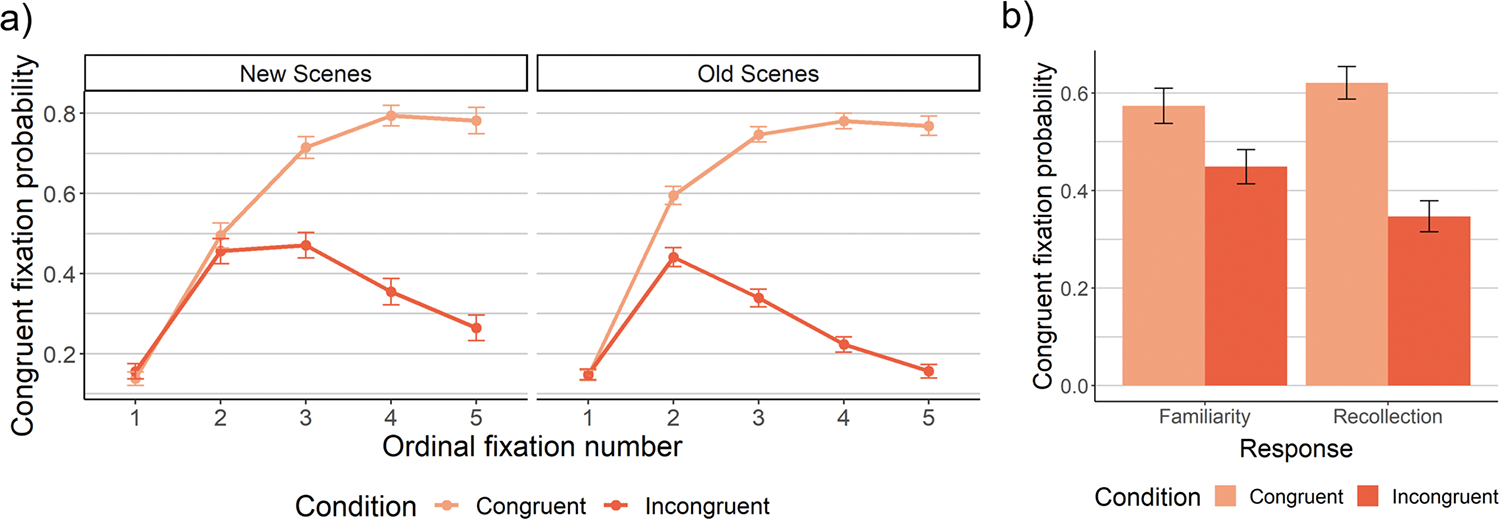
Effects of memory and semantic knowledge on the probability of fixating a congruent region. Memory – specifically recollection – increased first saccades to congruent regions for congruent scenes, but decreased them for incongruent scenes. (**A**) The probability that each fixation was made to a congruent region, by ordinal fixation number in a trial. Note that fixation 1 in this plot was the fixation on the central fixation cross (i.e., no voluntary eye movements had yet occurred). The analyses of the probability of the first saccade landing on a congruent region are focused on the saccade between fixation 1 and fixation 2. (**B**) The probability that the first saccade off the central fixation cross landed on a congruent region for recollection versus strength-matched familiarity. For both plots, the estimated marginal means and standard errors derived from the model are plotted

We next examined the likelihood that the first saccade upon search scene onset landed on a congruent region (Fig. 5). Neither memory overall (Eq. S1a), nor recollection (Eq. S2a), exhibited a main effect, *p*s > .30. Instead, memory interacted with congruency such that first saccades were most likely to land on congruent regions in old, congruent scenes *B* = −.49, *p =* .002 (Fig. 5a; Eq. S1b). In particular, recollection interacted with schema congruency, *B* = −.62, *p =* .004 (Eq. S2b), such that first saccades to congruent regions were reduced for incongruent scenes but increased for congruent scenes (Fig. 5b). This held when the distance from the saccade landing point to the target was covaried, *B* = −.62, *p =* .005 (Eq. S2b with distance covariate added), suggesting that this effect was not simply a byproduct of spatial memory or presence of the target in the region. No main effects or congruency interactions were found for familiarity (Eq. S3) or unconscious memory (Eqs. S4), *p*s > .34. These findings indicate that recollection uniquely interacted with semantic knowledge to influence initial eye movements by increasing saccades to congruent regions in congruent scenes, and decreasing them in incongruent scenes – and this was not solely a byproduct of spatial memory. Rather, it appears likely that participants recollected the scenes’ congruency condition and used this knowledge to constrain early search.

### Effects on eye movements throughout search

**Fig. 6:**
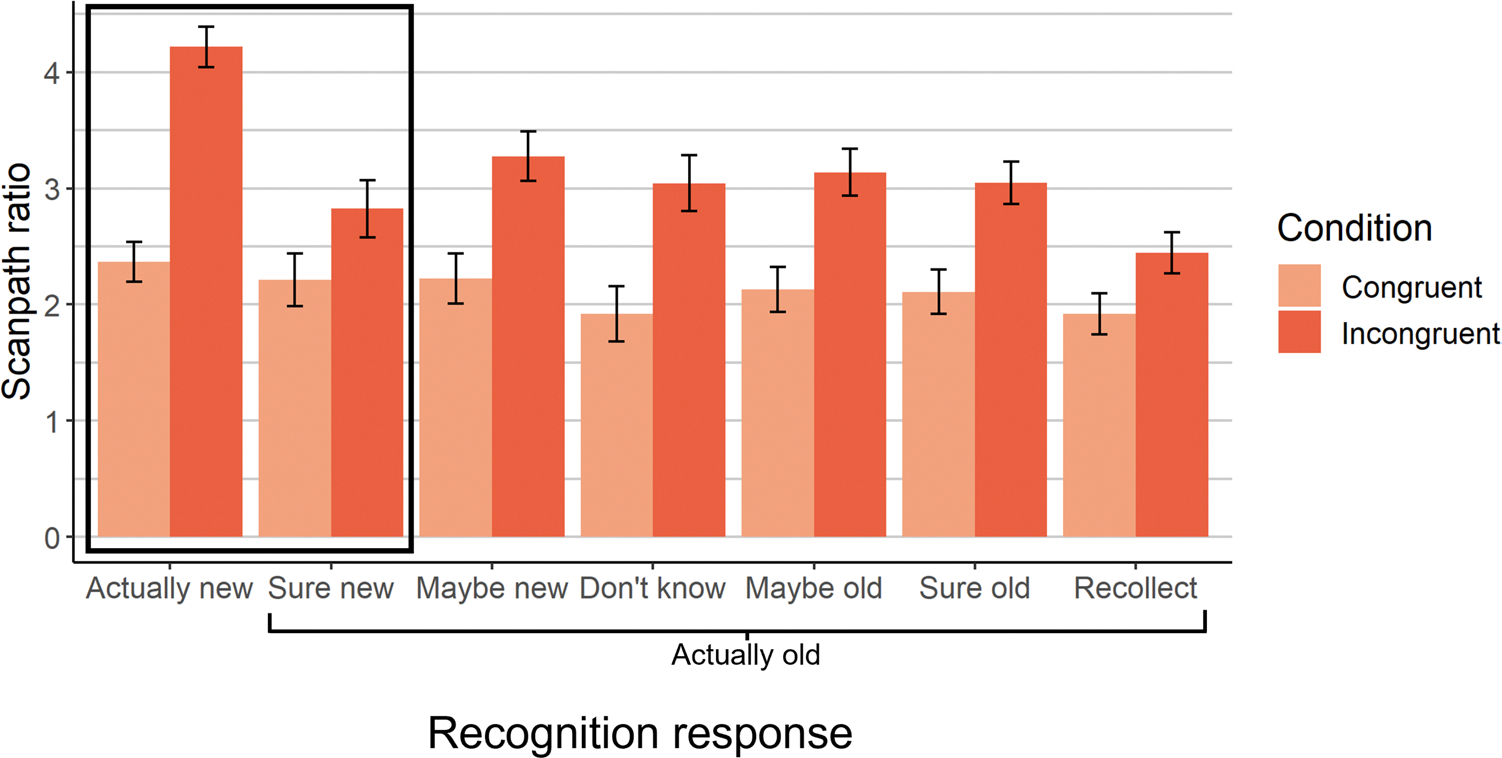
Scanpath efficiency over the course of the trial. Unconscious memory selectively reduced scanpath ratio for incongruent scenes. Note that higher scanpath ratio values denote less efficient search. The bars within the bounding box (Actually new and Sure new) represent the data used to assess the unconscious memory effects. The estimated marginal means derived from a linear mixed effects model with random effects of scene and subject are plotted, and the error bars represent the standard error of these estimated means from the model

We next examined eye movements throughout the search process. Both memory and semantic knowledge improved scanpath efficiency: Scanpath ratio was lower in old than new scenes, *SMD =* −.25, *p <* .0001 (Eq. S1a), and in congruent than incongruent scenes, *SMD =* −.46, *p <* .0001 (Fig. 6). Moreover, memory-driven improvements to scanpath ratio were larger for incongruent than congruent scenes, *SMD =* −.22, *p =* .0003 (for interaction; Eq. S1b), which parallels the reaction time findings. This effect held when controlling for scaling effects (and first saccade accuracy): Memory improved scanpath ratio by 29% in incongruent scenes, compared to 12.4% in congruent scenes, *SMD =* −.21, *p <* .0001 (Eq. S5).

Decomposing these effects, recollection improved scanpath ratio, *SMD =* −.20, *p <* .0001 (Eq. S2a), but did not interact with congruency to do so, *SMD =* −.11, *p =* .21, BF_10_=.21 (Eq. S2b). Familiarity did not exhibit any effects, *p*s>.41, BF_10_s<.08 (Eqs. S3). Unconscious memory improved scanpath ratio, *SMD =* −.23, *p =* .0003 (Eq. S4a), replicating prior findings (Ramey et al., 2019). That is, scanpath ratio was significantly better for high-confidence misses (i.e., “I’m sure it’s new” old scenes) than new scenes, indicating an experience-driven search performance improvement despite a lack of awareness for that experience. Unconscious memory interacted with congruency to influence scanpath ratio, *SMD =* −.27, *p =* .008 (Eq. S4b), such that unconscious memory significantly improved scanpath ratio in incongruent scenes, *SMD =* −.30, *p =* .0005, but not congruent scenes, *SMD =* −.11, *p =* .15, BF_10_ = .28. To confirm that these effects of unconscious memory occurred after the initial eye movement, we recalculated scanpath ratio after the first saccade (i.e., from the second fixation onward), and found that both the main effect of unconscious memory on scanpath ratio, *SMD =* −.24, *p =* .004, and its interaction with congruency, *SMD =* −.33, *p =* .02, held. The presence of this selective unconscious interaction with congruency, without any such effects of recollection or familiarity, indicates that the interaction between overall memory and schema congruency was driven by memory that was not accessible to awareness. This supports our prediction that unconscious memory, via its gradual effects on eye movements, primarily contributes to performance when semantic knowledge fails to successfully guide early eye movements to the target.

### Summary of results by memory type

To summarize the results for each type of memory: We found that overall memory (i.e., all old vs all new scenes, collapsed across responses) improved first saccade accuracy in that first saccades were aimed more directly towards the target. Accounting for scaling revealed that memory improved first saccade accuracy by a higher proportion for congruent than incongruent scenes. Overall memory interacted with congruency condition to modulate whether first saccades landed on congruent regions. Overall memory also improved search efficiency in the form of scanpath ratio, and primarily did so for incongruent scenes. Decomposing these effects by memory type, recollection improved first saccade accuracy and scanpath ratio, and did not exhibit interactions with congruency to do so. However, recollection interacted with congruency condition to modulate the likelihood of first saccades to congruent regions: Recollection increased the likelihood of congruent first saccades in congruent scenes, but decreased them in incongruent scenes. Familiarity strength did not influence any aspects of search performance. Unconscious memory did not influence first saccade accuracy or likelihood of first saccades to congruent regions. However, unconscious memory improved scanpath ratio, and interacted with congruency such that unconscious memory improved scanpath ratio for incongruent scenes but not congruent scenes. Overall, therefore, recollection appears largely responsible for the effects of memory on the first saccade, whereas unconscious memory appears largely responsible for the interactions between memory and congruency in influencing overall search efficiency.

## Discussion

We examined how semantic knowledge interacts with different types of episodic memory to guide attention during visual search. We manipulated the effectiveness of semantic knowledge guidance by placing target objects in schema-congruent or incongruent locations in scenes, and we examined different types of episodic memory by assessing confidence-based recognition memory for the background scenes. As hypothesized, unconscious memory improved search efficiency only when semantic knowledge failed to successfully guide early eye movements to the target (i.e., in incongruent scenes). We also found that recollection uniquely influenced early eye movements such that first saccade accuracy was higher for recollected than confidence-matched familiar scenes regardless of congruency. In contrast to our predictions, however, interactions between memory and semantic knowledge in guiding the first eye movement appeared to be synergistic rather than competitive: Proportionally, memory-driven improvements in first saccade accuracy for congruent scenes were more than twice as large as for incongruent scenes. Interestingly, familiarity-based memory had no detectable influence on search performance. Together, these results indicate that the dynamics of interactions between semantic knowledge and episodic memory in guiding attention critically depend on the type of episodic memory involved.

Our results provide a potential resolution to prior conflicting findings on the extent to which memory contributes to semantically relevant search. Specifically, we found that unconscious memory had little to no effect when semantic knowledge was effective, which supports the proposal that memory often does not influence semantically relevant search (Võ & Wolfe, 2012, 2013). In contrast, recollection consistently improved first saccade accuracy regardless of semantic knowledge effectiveness, which supports the opposing proposal that memory consistently benefits semantically relevant search (Hollingworth, 2012; Howard et al., 2011). It is therefore possible that studies in which memory exerted little effect on search had low rates of recollection, such that unconscious memory was the primary type of (search-relevant) memory available. (Note that familiarity would be able to support object recognition, but the current findings and previous findings indicate it does not influence search; Ramey et al., 2019.) Differences in the type of memory available across studies could be driven by differences in the distinctiveness and memorability of targets and distractors within the stimuli used (Hunt, 1995; Rust & Mehrpour, 2020). Overall, our findings can reconcile the conflicting proposals from prior work: The most accessible or rapidly effective form of information is prioritized in search, and memory does consistently influence search alongside semantic knowledge provided that it is a type of memory that is accessible and rapidly effective.

More broadly, the present findings point to a new potential theory of attentional guidance, which we term *rational integration*: Different sources of information, in this case semantic knowledge and episodic memory, may be rationally combined to guide attention. That is, a growing body of work indicates that when making memory decisions—for example, remembering where an object was in a scene – memory and semantic knowledge are rationally combined according to their strength or precision (Duffy et al., 2006; Hemmer & Persaud, 2014; Hemmer & Steyvers, 2009; Huttenlocher et al., 1991; Macias & Persaud, 2024; Ramey et al., 2022). The present findings indicate that this type of process may play out in attentional guidance as well. When only unconscious memory was available, the most effective form of guidance available – semantic knowledge – was prioritized. When more precise recollection was available, it was instead prioritized alongside semantic knowledge. In fact, recollection appeared to be integrated with semantic knowledge to enhance performance, such that there was a proportionally larger memory benefit for congruent scenes. It could be, for example, that recollection constrains viewing to congruent surfaces in the correct general region. Moreover, rational integration can account for studies finding no effects of memory on search even when semantic knowledge is unavailable (Kunar et al., 2008; Wolfe et al., 2000): Other stimulus properties, besides semantic knowledge, are likely prioritized above memory to guide search in those contexts. Rational integration of different sources of information could also extend beyond memory: For example, task goals may modulate how visual salience is leveraged to guide attention (Gaspelin et al., 2017; Geng, 2014).

The present findings may also help inform our understanding of how memory and semantic knowledge interact more broadly. Semantic knowledge exhibits robust interactions with episodic memory across a variety of domains (Berens et al., 2020; Ghosh & Gilboa, 2014; Greve et al., 2019; Macias & Persaud, 2024; Tompary & ThompsonSchill, 2021; van Kesteren et al., 2012; Voss et al., 2012), and different episodic processes interact differently with schemas at retrieval (Lampinen et al., 2001; Prull, 2015; Ramey et al., 2022). For example, in prior work using the same stimuli and study-phase search task as the present study, recollection abolished effects of semantic knowledge on spatial recall accuracy for the target object locations (Ramey et al., 2022; Ramey & Zabelina, 2024). The present findings, along with additional recent findings, help to understand the mechanisms underlying this effect. Specifically, in a recent study, recollection increased the likelihood of recalling an object as having been in a congruent region for congruent scenes, and decreased that likelihood for incongruent scenes – even when controlling for spatial accuracy (Ramey et al., 2024). We found exactly this effect with respect to congruent first fixations in the present study. Therefore, recollection may improve memory performance in congruent and incongruent scenes in distinct ways that can render them largely equivalent in accuracy: suppressing schema-based responding for incongruent scenes and amplifying it for congruent scenes.^3^ Together, therefore, these results indicate that recollection may be integrated with semantic knowledge in nuanced ways, rather than suppress its use altogether. This suggests that current theories of how semantic knowledge and memory are rationally combined may need to be updated to account for potentially synergistic dynamics.

There are limitations to this study that should be noted. First, in the present study, as well as in the most relevant prior studies, the scene was visible prior to commencing search for the target – in our case, only a preview of the background scene without the target was shown before search. Given that the process of recognition itself, in particular via recollection, is relatively slow (~800ms for recollection; Yonelinas, 2002; Zimmer & Ecker, 2010), it is likely that if participants were required to simultaneously conduct visual search while trying to recognize the scene, recollection would not influence the first eye movement. Rather, our results indicate that when both recollection and semantic knowledge are available, as in most real-world situations (i.e., we do not typically experience instant, unexpected scene onsets that we have to immediately search through), they can both guide attention immediately upon commencing search. A second consideration is that the target-absent preview we used may have influenced rates of recollection and/or familiarity relative to target-present previews. Third, given that half of the scenes were incongruent, participants may have learned to down-weight the use of schema information (see Macias & Persaud 2024). Thus, the effects of semantic knowledge on attention may have been weaker than in the real world.

In sum, the present findings demonstrate that interactions between semantic knowledge and episodic memory in guiding attention depend critically on the type of episodic memory involved: Recollection improved early eye movements alongside semantic knowledge, whereas unconscious memory improved later efficiency only when semantic knowledge failed. Moreover, these results suggest that apparently competing influences on our attention may be resolved via rational integration – that is, cooperation and combination to optimize performance – rather than competition to dominate attention.

## Supplementary Material

Supp1

**Supplementary information** The online version contains supplementary material available at https://doi.org/10.3758/s13423-025-02686-6.

## Figures and Tables

**Table 1:** Trial counts for each memory response for old and new scenes

	“Sure new”	“Maybe new”	“Don’t know”	“Maybe old”	“Sure old”	“Recollect old”

Old	446	547	411	683	825	988
New	428	307	132	163	86	54

*Note.* Note that there were more old than new scenes overall

## Data Availability

The data are being used for a modeling study and will be made publicly available once all studies using it are completed and published; data and code are available upon request.

## Footnotes

1 Our definition of unconscious memory is a change in behavior due to prior experience with an item even when participants are highly confident that they do not have prior experience with the item. (Our definition does not address the extent to which this is based on the same underlying process as weak conscious memory, or comprises a distinct process.)

2 However, it is possible that familiarity could influence semantically relevant search in particular. A selective effect of familiarity on semantically relevant search would be in line with prior findings that familiarity strength and local semantic meaning exhibit a tradeoff in predicting attention during free viewing (Ramey et al., 2020), along with findings that familiarity partially suppresses semantic influence on memory decisions (Ramey et al., 2022). Moreover, a selective role of familiarity in semantically relevant (but not irrelevant) search would support theories that familiarity largely depends on semantic knowledge (i.e., conceptual processing; Wagner & Gabrieli, 1998).

3 Another possibility is that differences in memory *encoding* by congruency influenced the present effects. For example, if recollection itself is better for congruent scenes, this could produce the presently observed synergistic effects. However, this is unlikely for at least three reasons. First, incongruent information typically leads to more recollection and better memory overall than congruent information, likely due to distinctiveness and/or novelty effects (Greve et al., 2019; Lampinen et al., 2001; Ramey et al., 2024; van Kesteren et al., 2012). Moreover, in the present study, there were no significant effects of congruency on recollection or familiarity estimates, perhaps due to assessment of recognition for the background scene rather than the congruency-related information itself. Second, we conditionalized all analyses on the type and strength of memory that resulted from encoding; thus, the extent to which different congruency conditions might produce different levels of memory strength, and recollection versus familiarity, was controlled for. Third, in prior work, recollection produced equivalently precise spatial memory for congruent and incongruent scenes (Ramey et al., 2022; Ramey & Zabelina, 2024). Moreover, for other types of memory judgments, memory is more precise for incongruently than congruently located objects (Wynn et al., 2020) – which is in the opposite direction of what could explain the present findings. Therefore, the evidence thus far suggests that our findings likely do not reflect encoding effects, but future studies are needed to determine whether encoding mechanisms may also be involved.
