# Supplementary material for: Episodic memory and semantic knowledge interact to guide eye movements during visual search in scenes: Distinct effects of conscious and unconscious memory
Source: Psychon Bull Rev. Author manuscript; Available in PMC 2025 Jul 18. (PMC12273590; doi:10.3758/s13423-025-02686-6)
Supplement: Supplementary file 1 [file NIHMS2093869-supplement-Supp1.docx]

Supplementary Materials for Ramey, Henderson & Yonelinas (2025)

*Psychonomic Bulletin & Review*

**Supplementary Results**

	In terms of effects of different memory processes on search speed (time to fixate the target), both recollection, *SMD*=-.14, *p*=.003, and unconscious memory, *SMD*=-.25, *p*<.0001, improved search speed. Recollection did not significantly interact with congruency to do so, *SMD*=-.16, *p*=.07, but unconscious memory did in a manner analogous to its effects on scanpath ratio, *SMD*=-.23, *p*=.028. Familiarity strength did not influence search speed, β=.03, *p*=.09, nor did it interact with congruency, β=.005, *p*=.89.

The descriptive statistics for the response frequencies by participant are as follows: “(1) sure new” *M*=22.4, *SD*=12.2, “(2) maybe new” *M*=21.8, *SD*=10.2, “(3) don’t know” *M*=13.9, *SD*=7.8, “(4) maybe old” *M*=21.7, *SD*=10.1, “(5) sure old” *M*=23.4, *SD*=11.8, “(6) recollect old” *M*=26.7, *SD*=18.9. See Figure S1 for the histogram of response frequencies by condition.

**Figure S1** 

*Recognition Response Frequencies for Old and New Congruent and Incongruent Scenes*

*Note*. The correspondence between recognition response number (1-6) and their associated responses are outlined in the preceding paragraph.

**Model Equations**

Note that random slopes were tested for every analysis and all effects (or lack thereof) held. The outcome measures are defined in the results section; they include reaction time, first saccade accuracy, probability of the first saccade landing on a congruent region, and scanpath ratio. Note that landing on a congruent region was a binary outcome and a logistic mixed effects model was therefore used for those analyses.

***Equation S1: Effects of overall memory***

Data included: all scenes

**Eq. S1a (main effect):** Eye movement measure ~ (old vs new) + (1| subject) + (1| stimulus)

**Eq. S1b (interaction with schema congruency):** Eye movement measure ~ (old vs new) * (schema congruency) + (1| subject) + (1| stimulus)

***Equation S2: Effects of recollection***

Data included: “sure old” and “recollect old” old scenes

**Eq. S2a (main effect):** Eye movement measure ~ (response: “sure old” vs “recollect old”) + (1| subject) + (1| stimulus)

**Eq. S2b (interaction with schema congruency):** Eye movement measure ~ (“sure old” vs “recollect old”) * (schema congruency) + (1| subject) + (1| stimulus)

***Equation S3: Effects of familiarity***

Data included: “sure new” through “sure old” old scenes

**Eq. S3a (main effect):** Eye movement measure ~ (response: continuous “sure new” through “sure old”) + (1| subject) + (1| stimulus)

**Eq. S3b (interaction with schema congruency):** Eye movement measure ~ (response: continuous “sure new” through “sure old”) * (schema congruency) + (1| subject) + (1| stimulus)

***Equation S4: Effects of unconscious memory***

Data included: “sure new” old scenes and new scenes

**Eq. S4a (main effect):** Eye movement measure ~ (“sure new” old vs new) + (1| subject) + (1| stimulus)

**Eq. S4b (interaction with schema congruency):** Eye movement measure ~ (“sure new” old vs new) * (schema congruency) + (1| subject) + (1| stimulus)

***Equation S5: Correcting for scaling***

An old/new difference score was created for each eye movement, so all scenes were included.

Difference score ~ (schema congruency) + (1| subject) + (1| stimulus)

**Pseudo-R**^**2**^ **for Key Effects**

| Model | Eye movement measure | Marginal R^2^ | Conditional R^2^ |
| --- | --- | --- | --- |
| S1b (overall memory) | First saccade accuracy | 0.04 | 0.11 |
| S1b (overall memory) | Congruent first saccades | 0.018 | 0.37 |
| S1b (overall memory) | Scanpath ratio | 0.07 | 0.26 |
| S2a (recollection) | First saccade accuracy | 0.003 | 0.05 |
| S2b (recollection) | Congruent first saccades | 0.04 | 0.24 |
| S4a (unconscious) | Scanpath ratio | 0.01 | 0.23 |
| S4b (unconscious) | Scanpath ratio | 0.08 | 0.3 |
